# Enhanced Antioxidant and Cytotoxic Potentials of Lipopolysaccharides-Injected *Musca domestica* Larvae

**DOI:** 10.3390/pharmaceutics12111111

**Published:** 2020-11-19

**Authors:** Islam El-Garawani, Hesham El-Seedi, Shaden Khalifa, Islam H. El Azab, Marwa Abouhendia, Shaymaa Mahmoud

**Affiliations:** 1 Department of Zoology, Faculty of Science, Menoufia University, Menoufia 32511, Egypt; marwaabohendia@science.menofia.edu.eg (M.A.); dr.shaymaahussein@science.menofia.edu.eg (S.M.); 2 Department of Molecular Biosciences, The Wenner-Gren Institute, Stockholm University, S-10691 Stockholm, Sweden; shaden.khalifa@su.se; 3 Chemistry Department, Faculty of Science, Menoufia University, Menoufia 32511, Egypt; 4 International Research Center for Food Nutrition and Safety, Jiangsu University, Zhenjiang 212013, China; 5 Chemistry Department, College of Science, Taif University, P.O. Box 11099, Taif 21944, Saudi Arabia; i.helmy@tu.edu.sa; 6 On Leave from Chemistry Department, Faculty of Science, Aswan University, Aswan, P.O. Box 81528, Aswan 81528, Egypt

**Keywords:** *M. domestica* larva, hemolymph, lipopolysaccharides, antioxidant, cytotoxicity, superoxide dismutase 1, glutathione, malondialdehyde

## Abstract

The usage of insects as a sustainable and functional natural products resource is a new promise in complementary and alternative medicine. The present study aimed to investigate the ability of *Musca domestica* (housefly) larval hemolymph (insect blood) to display the enhanced in vitro antioxidant and cytotoxic effects. The oxidative stress (OS) was elicited by inducing lipopolysaccharides (LPS) treatment as an exogenous stressor. Determination of superoxide dismutase 1 (SOD1), glutathione (GSH), malondialdehyde (MDA) and total antioxidant capacity (TAC), and mRNA and protein expressions of SOD1, was investigated as confirmatory markers of oxidative stress induction. Cytotoxicity on cancerous MCF-7 and normal Vero cells were also evaluated using an MTT assay at 24 h post-injection. The injection of LPS induced a significant (*p* < 0.05) increase in SOD, GSH and TAC, whereas, the MDA was diminished. Hemolymph was collected from normal and treated larvae after 6, 12 and 24 h. The *M. domestica* superoxide dismutase (*Md*SOD1) transcripts were significantly (*p* < 0.05) upregulated 6 and 12 h post-treatment, while a significant downregulation was observed after 24 h. Western blot analysis showed that *Md*SOD1 was expressed in the hemolymph of the treated larvae with an increase of 1.2 folds at 6 and 12 h and 1.6 folds at 24 h relative to the control group. LPS-treated larval hemolymphs exhibited significant cytotoxicity with respect to the untreated ones against MCF-7 while Vero cells showed no cytotoxicity for both hemolymphs. The DPPH free radical scavenging activity was examined and a significant antioxidant potential potency was observed at 6 h (50% maximal inhibitory concentration (IC_50_): 63.3 ± 3.51 µg/mL) when compared to the control *M. domestica* larval hemolymph (IC_50_: 611.7 ± 10.41 µg/mL). Taken together, *M. domestica* larval hemolymph exhibited enhanced antioxidant and consequently increased cytotoxic capacities under stressed conditions.

## 1. Introduction

The biological applications of various natural products were the interest of researchers decades ago. Natural product extracts can exert chemoprotective [1,2], antigenotoxic [3], antihelminthic [4] and anticancer potency [5,6,7,8,9]. Similarly, several bioactive compounds were identified from insects and have elicited significant bioactivities including anticancer [10,11,12], antihyperlipidemic [13], antiulcer [14], cardioprotective [15], antidiabetic [16], antimicrobial [17,18] and anti-inflammatory [19,20,21]. In this essence, insects are already well-known to represent a valid source of novel proteins, minerals, vitamins, and fatty acids, however, the role of their bioactive ingredients is scarcely studied [22].

The housefly, *Musca domestica* is a domestic medical and veterinary pest and the most common among domestic flies accounting for about 90% of all flies in human habitation [23]. It can cause serious diseases like typhoid fever and cholera by carrying the pathogenic agent. However, to the best of our knowledge, few biological studies have reported that the housefly larvae have antiviral and antitumor [24], antibacterial [25] and antimalarial activity [26]. The housefly larvae have been used clinically to treat malnutrition, pressure ulcers, osteomyelitis, eczema and herpes simplex viral infection. The housefly larval extract has been reported as an antitumor agent [27,28], and they are also used in combination with other drugs against gastric cancer [29]. The chemical composition of *M. domestica* hemolymph is very complex, consists mainly of antibacterial proteins and carbohydrates, such as the antimicrobial peptides, lysozyme and agglutinin [30,31]. There are increasing interests of investigating the structures and functions of the insects’ active ingredients particularly those with antioxidant capacities. Insects have evolved a complex and efficient network of enzymatic antioxidant systems for their self-protection against reactive oxygen species (ROS) [32]. This self-defense cascade is carried out by free radical scavenging activity and the repair of the damaged biomolecules essential for life [33]. The considerable protein content of insects supports their nutritional value [34]. Therefore, bioactive peptides with beneficial biological activity [35] such as anti-inflammatory, antioxidant, antihypertensive and even hypocholesterolemic can be a good source of nutritional value [36,37,38,39] via stimulation of GST and CAT antioxidant enzymes and DPPH radical scavenging activities such as *Gryllodes sigillatus* hydrolysates [35] and the aqueous extract of *Calliptamus italicus*, *Bombyx mori*, *Vespa affinis* and *Acheta domesticus* [40,41]. A series of investigations confirmed the potential of several chemicals; H_2_O_2_ [42] and biological compounds [43] including pesticides and prooxidant allelochemicals to elicit oxidative stress (OS) conditions in the insect models [44], posing a serious challenge to the insect species.

Lipopolysaccharides (LPS), bacterial endotoxins, are the main component of the outer membrane of Gram-negative bacteria, which is known to induce oxidative stress in MAC-T cells in vitro [45] and oxidative damage to the mammary gland in vivo [46]. Moreover, it caused liver and neurological damage, diabetes and gut chronic inflammation [47,48]. LPS was used to induce oxidative stress in insects [49,50,51]. However, it also stimulated anti-inflammatory [52] and immune responses [51,53].

Constantly, normal metabolic processes in the body produce free radicals, but the imbalance between the generation and the ability of cells to neutralize those results in oxidative stress. The occurrence of oxidative stress has been implicated in the pathogenesis of chronic diseases, such as arthritis, diabetes, cancer, stroke, myocardial infarction and the degenerative aliments associated with aging, including Parkinson’s and Alzheimer’s diseases [54,55,56,57]. Consumption of foods rich in antioxidants plays an essential role in the prevention of these diseases as the dietary antioxidant and anti-inflammatory peptides have protective effects against ROS and may contribute to a significant reduction of the level of oxidative stress [35,58,59,60]. Both clinical and experimental studies have indicated the advantages of antioxidant supplementation as a therapeutic tool to treat oxidative stress-related health disorders [61,62] and cancer, its initiation and progression [63]. However, the antioxidant activities of larval hemolymph have not been examined yet. This study, therefore, aimed to evaluate the efficacy of the larval hemolymph of *M. domestica* as a natural antioxidant, for the first time, introducing it as a potential therapeutic and food supplement under both normal and activated stressful conditions.

## 2. Materials and Methods

### 2.1. Insect Rearing

*M. domestica* flies were prepared, colonized, reared to the adult stage and maintained in the Zoology Department insectary, Faculty of Science, Menoufia University. The laboratory conditions were kept to 26 ± 1 °C; photoperiod: 14 L:10 D and relative humidity: 60% ± 10%. A 10% sucrose solution was supplied to the adults while bovine meat was the feeding subject for the larvae [64]. This study set-up was designed and planned following the approval of the institutional research committee, Menoufia University, Shebin El-Kom, Egypt (MUFS-F-GE-7-20) on the ethical standards and in agreement with the declarations of Helsinki (1964) and the later amendments.

### 2.2. Oxidative Stress Induction

To assess the antioxidant effect of the larval defense system, the newly molted third instar larvae with relatively uniform age and weight (40 ± 5 mg) were stressed by injecting 1 μg/larva, into the hemocoel, of lipopolysaccharides (LPS, *Escherichia coli* O111:B4, L3012-5MG-PW) purchased from Sigma Chemical Company (St. Louis, MO, USA) as described by Parusel et al. [50]. The injection was done to the larvae using a sterile, thin-needled microsyringe.

### 2.3. Hemolymph Collection

Hemolymph (1 µL/larva) was pooled by cutting off the anterior tip of the larvae with sterile fine scissors in a prechilled eppendorf containing few crystals of phenylthiourea to prevent melanization [42]. Hemolymph samples were collected at 6, 12 and 24 h post-injection and from untreated larvae as controls.

### 2.4. Determination of Antioxidant Enzymes Activity

#### 2.4.1. Superoxide Dismutase (SOD)

The activity of SOD was determined by the inhibition of pyrogallol auto-oxidation. The inhibition is directly proportional to the activity of SOD in the tested samples. Changes in absorbance at 420 nm were recorded every minute for 3 min, using a spectrophotometer (Milton Roy, Spectronic 1201, Houston, TX, USA) [58,65].

#### 2.4.2. Glutathione (GSH)

The method of Ellman [66] was adopted to test glutathione, where the reduction of Ellman’s reagent (5,5′-dithio-bis (2-nitrobenzoic acid)) is the indicator. The intense yellow color of the formed nitromercaptobenzoic acid distinguishes the reduction and can be measured colorimetrically at 412 nm (Milton Roy spectrophotometer, Spectronic 1201, Houston, TX, USA).

#### 2.4.3. Malondialdehyde (MDA)

MDA colorimetric determination can be measured spectrophotometrically with double wavelength at 535 and 520 nm (Milton Roy spectrophotometer, Spectronic 1201, Houston, TX, USA). The developed pink color is a visual sign to highlight the reduction and to avoid interference following the method of Mihara and Uchiyama [67].

### 2.5. Protein Electrophoresis (SDS-PAGE)

Total protein concentrations were extracted from the samples using a Tris-buffer system prior to the colorimetrical control using the Bradford Protein Assay Kit (ab102535, Abcam, Cambridge, UK). The hemolymph total soluble proteins were qualitatively analyzed of employing sodium dodecyl sulphate-poly acrylamide gel electrophoresis (SDS-PAGE) as described by Laemmli [68]. Briefly, protein samples of equal amounts (25 µg) were mixed with SDS sample buffer and boiled for 5 min, then ice-cooled for 7 min followed by SDS-polyacrylamide gel (15% resolving gel) and the vertical electrophoresis unit (Cleaver, UK) for the final ingredients separation. To estimate the molecular weights of the separated bands, a prestained molecular weight marker of the low range 180–10 kDa (Sigma, St. Louis, MO, USA) was loaded.

### 2.6. Western Blot Analysis

Western blot analysis was performed as described by Burnette [69], where the resolved electrophoresed proteins were subjected to 15% SDS-polyacrylamide gel before being added onto polyvinylidene fluoride (PVDF) membranes (Bio-Rad, Hercules, CA, USA) for 30 min using a Semi-dry Electroblotter (Bio-Rad, Hercules, CA, USA) at 2.5 A and 25 V for 30 min. The blocking was done with the help of 5% nonfat dry milk in TBS-T and kept at room temperature for two hours, in order to reduce the non-specific protein binding between the membrane and the antibodies. The following step was the incubation at 4 °C with each primary antibody overnight. The primary antibodies against SOD1 (rabbit polyclonal anti-SOD1, ab183446, Abcam, Cambridge, UK) and β-actin (rabbit polyclonal anti-β-actin, ab8227, Abcam, Cambridge, UK) proteins were prepared following the supplier’s instructions. The blots were then washed three times (10 min each) with Tris-Buffered Saline Tween (TBS)-T, incubated with the corresponding horse-radish peroxidase-linked secondary antibodies (Dako, Glostrup, Denmark) at room temperature for another hour, followed by three times wash. The chemiluminescent Western ECL substrate (Perkin Elmer, Waltham, MA, USA) was then added in accordance to the manufacturer’s recommendation. The chemiluminescent signals were captured using a Chemi Doc imager (Bio-Rad, Hercules, CA, USA). Protein bands were visualized using a Bio-Rad Chemi Doc Imager (Bio-Rad, Hercules, CA, USA), the band intensities were then calculated and normalized to the β-actin and analyzed by Gel Doc Go System (Bio-Rad, Hercules, CA, USA). The expected molecular weight of SOD1 protein was 18 kDa.

### 2.7. Quantitative Real-Time PCR (qPCR) for the MdSOD1 Gene

The changes in the expression pattern of *M. domestica* superoxide dismutase (*Md*SOD1) were measured following LPS injection according to the method of Wang et al. [70]. Larvae from the injected and control groups were separately collected at 6, 12 and 24 h after the injection for RNA extraction. The mRNA expression of the *Md*SOD1 at each time point was measured by qRT-PCR (Applied Biosystems™ 7500 Real-Time PCR System, Foster City, CA, USA). Briefly, total RNA was extracted using the Trizol reagent (Thermo Fisher Scientific, Austin, TX, USA) and genomic DNA was digested with RNA-free DNase. The specific primers were designed using primer3 software based on the sequences deposited on GenBank Database (Table 1). β-actin gene of *M. domestica* was used as an endogenous house-keeping gene. The qPCR was performed on Biosystem step one plus instrument using Maxima SYBR Green/ROX qPCR Master Mix (SABiosciences™, Applied Biosystems, Foster City, CA, USA). Reaction mixtures were incubated for 10 min at 95 °C, followed by 40 cycles of 15 s at 95 °C, 60 s at 60 °C and finally 60 s at 72 °C, melting curve from 70.0 to 95.0 °C, read every 0.3 °C and held for 10 s in a final volume of 20 µL. The relative expression ratios of *Md*SOD1 were calibrated against the control samples.

### 2.8. In Vitro Anticancer Activities

#### 2.8.1. Maintenance of Cell Lines

Human breast adenocarcinoma (MCF-7) and normal African green monkey kidney (Vero) cell lines were purchased from the Holding Company for Biological Products and Vaccines (VACSERA), Giza, Egypt.

Cell lines were maintained and cultured in Dulbecco’s modified Eagle’s medium (DMEM) supplemented with 10% fetal calf serum, 100 μg/mL streptomycin and 100 U/mL penicillin. Cells were incubated at a density of 2 × 10^4^ cells/cm^2^ in T25 culture flasks in a humidified 5% CO_2_ incubator adjusted to 37 °C. The medium was changed every 48 h. The cells were allowed to adhere up to 75% confluence then harvested after trypsinization (0.025% trypsin and 0.02% EDTA) and washed twice with phosphate-buffered saline (PBS). All experiments were done in triplicates and controlled under an inverted microscope. All reagents and media were purchased from Lonza supplier, Caioro, Egypt.

#### 2.8.2. Cytotoxicity Assay Using (3-(4,5-Dimethylthiazol-2-yl)-2,5-diphenyltetrazolium Bromide Dye (MTT)

The hemolymph cytotoxicity towards MCF-7 cells and the Vero normal kidney cells was assessed. Cells were seeded at a density of 1 × 10^4^ cells/well for 48 h in a 5% CO_2_ humidified incubator at 37 °C until reaching 70% confluence. A 10 μL of MTT dye was added to each well (final concentration 0.5 mg/mL) at 24 h post-injection and for 4 h, followed by 100 μL of MTT destaining solution (acidified isopropanol) on a shaker for 15 min. The optical density was measured at 550 nm with the microplate reader (RADIM SEAC Sirio S, Pomezia, Italy) to determine the number of viable cells. The percentage of inhibition was calculated in accordance to the following equation:

% Cell inhibition = (1 − OD (absorbance) test/OD Control) × 100(1)

The inhibition curve was performed and the 50% maximal inhibitory concentration (IC_50_) was calculated using Graphpad Prism 8 software (Graphpad Co., San Diego, CA, USA, 2019).

### 2.9. Determination of Antioxidant Activity In Vitro

#### 2.9.1. DPPH Assay

DPPH (2,2-diphenyl-1-picryl-hydrazil) radical scavenging assay is a standard in vitro antioxidant test [71]. An aliquot of 100 uL of the control and treated hemolymph samples were added to 1 mL of the freshly prepared methanol solution of DPPH (0.004%). DPPH solution stored at 10 °C in the dark was aliquoted to different concentrations between 0 and 640 μg/mL. Absorbance values were recorded immediately with a UV–visible spectrophotometer (Milton Roy, Spectronic 1201, Houston, TX, USA). The decrease in absorbance was determined and particularly at 515 nm. Ascorbic acid was used as a reference, and the IC_50_ was calculated using a logarithmic regression curve. All the experiments were done in three replicates. The percentage inhibition (PI) of the DPPH radical was calculated according to the formula:

PI = [(*A*_C_ − *A*_T_)/*A*_C_] × 100 (2)

where *A*_C_ = Absorbance of the control at t = 0 min and *A*_T_ = absorbance of the sample + DPPH at t = 16 min.

#### 2.9.2. Total Antioxidant Capacity (TAC)

Total antioxidant capacity (TAC) kit (ab65329, Abcam, Cambridge, UK) was administrated according to the manufacturer’s instructions, where the Cu^2+^ ion is converted to Cu^+^ by antioxidants (both small molecule and protein). To confirm the total antioxidant capacity of the small molecule antioxidants solely, protein mask was added and prevented Cu^2+^ reduction. The reduced Cu^+^ ion is chelated with a colorimetric probe giving a broad absorbance peak around 570 nm, proportional to the total antioxidant capacity. Trolox (6-hydroxy-2,5,7,8-tetramethylchromane-2-carboxylic acid), a water-soluble tocopherol analogue, was used as the standard.

### 2.10. Data Analysis

Results were representing three independent experiments and expressed as the mean ± SD. A one-way analysis of variance (ANOVA) determined the statistical significance, using IBM SPSS software version 21.1 (IBM, New York, NY, USA, 2019). The values with *p* < 0.05 were considered significant and *p* < 0.01 high significant.

## 3. Results

### 3.1. Antioxidant Enzymes Activity

**Figure 1:**
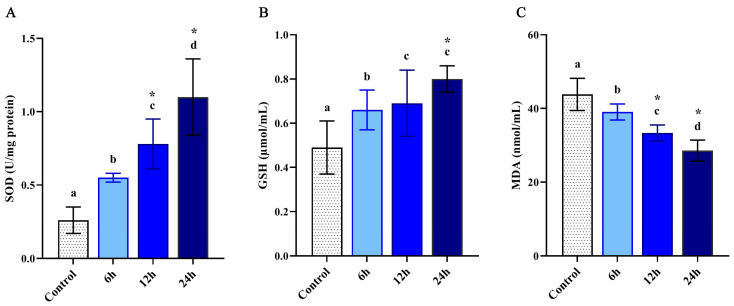
The activity of SOD (**A**), GSH (**B**) and MDA (**C**) of the control and injected *M. domestica* larval hemolymph at 6, 12 and 24 h post-injection with LPS. Different letters indicate significant difference (*p* < 0.05). * indicate high significance with respect to control (*p* < 0.01). Values were expressed as mean ± SD. The experiments were done in triplicate (*n* = 3).

In order to confirm the activation of the larval antioxidant defense system, SOD, GSH and MDA were investigated colorimetrically in the control and LPS-treated larvae. Results revealed a significant increase of SOD and GSH activities (*p* < 0.05) when compared with control (Figure 1A,B). The promotion of the antioxidant response was observed in a timely fashion corresponding to 6, 12 and 24 h post-injection and as a response to LPS injection. Whereas, the MDA levels significantly decreased in a time-dependent pattern (Figure 1C).

### 3.2. Altered Levels of MdSOD1 Protein

**Figure 2:**
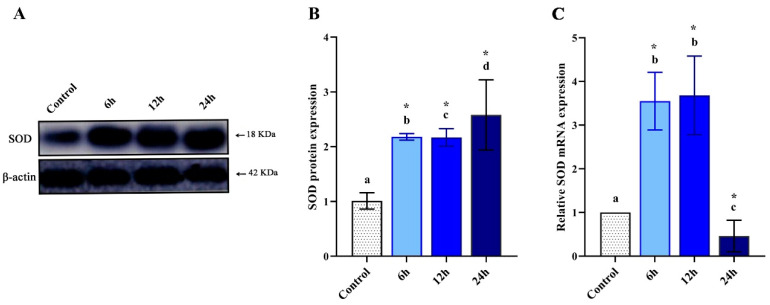
Changes in *Md*SOD1 expression of control and LPS-treated *M. domestica* larval hemolymph. (**A**) The representative photograph of protein expression analyzed by Western blotting. (**B**) A graph shows the analysis of protein bands (Gel Doc Go System, Bio-Rad, Hercules, CA, USA). (**C**) A graph shows the relative *Md*SOD1-mRNA expression analyzed by qRT-PCR. B and C, different letters indicate significant difference (*p* < 0.05). * indicate high significance with respect to control (*p* < 0.01). Data were presented as mean ± SD. The experiments were done in triplicate (*n* = 3).

Changes in *Md*SOD1 protein levels were detected in hemolymph of control and LPS-treated larvae at different intervals using Western blot analysis (Figure 2A). Results revealed a significant (*p* < 0.05) increase in the protein expression in the larvae hemolymph at all the tested time points with respect to the control. The increase was estimated to 1.2 folds for 6 and 12 h and 1.6 folds for 24 h relative to the untreated group (Figure 2B).

### 3.3. Altered Expression of MdSOD1 mRNA

In order to quantify the transcriptional levels of *Md*SOD1, mRNA levels were assessed using qRT-PCR. The LPS-treated larvae showed significant upregulation of *Md*SOD1 gene (*p* < 0.05) at 6 and 12 h post-injection (2.7 and 2.6 folds respectively, relative to the control). However, the 24 h interval showed a significant downregulation (*p* < 0.05) of the gene expression (Figure 2C).

### 3.4. Cytotoxicity of the M. domestica Larval Hemolymph

**Figure 3:**
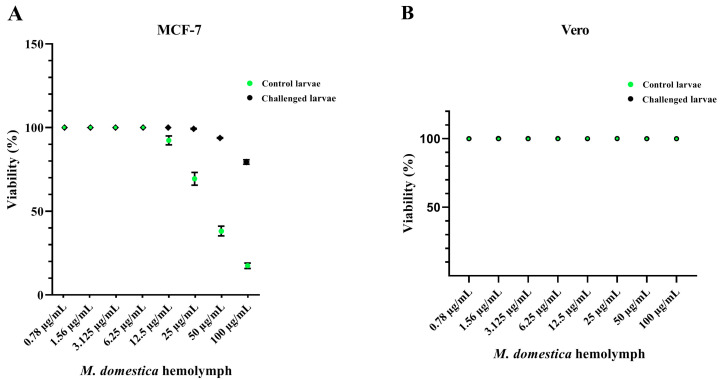
The viability of control and LPS-treated *M. domestica* larval hemolymph on MCF-7 (**A**) and Vero (**B**) cell lines using the MTT assay. The incubation with serial concentrations (0.78–100 µg/mL) of the tested larval hemolymphs was done in triplicates. Data were represented as (mean ± SD) of three independent experiments.

In order to assess the enhancement of *M. domestica* larval hemolymph’s anticancer potency, the cytotoxic effect of control and LPS-treated larval hemolymphs was investigated against MCF-7 and Vero cells using the MTT assay at 24 h post-injection. Figure 3A revealed that untreated larval hemolymph exhibited less cytotoxicity than the injected ones towards the cancer cells (MCF-7). About 79.2% ± 0.6% and 17.5% ± 1.68% viable MCF-7 cells were seen when hemolymph was applied up to 100 μg/mL in control and LPS-injected larval hemolymph respectively. The IC_50_ was 41.2 ± 2.75 μg/mL for the hemolymph of the injected larvae, while the control larval hemolymph revealed the IC_50_ at 100 μg/mL. However, no cytotoxicity was observed on Vero normal cells up to a concentration of 100 µg/mL in control and LPS-injected groups (Figure 3B).

### 3.5. DPPH Radical Scavenging Assay

**Figure 4:**
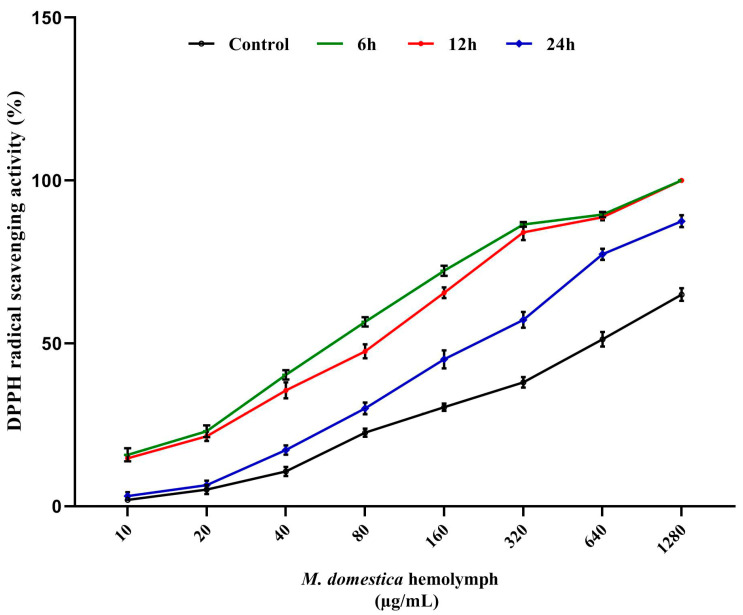
DPPH radical scavenging activities of *M. domestica* larval hemolymph. The graph is obtained by plotting different concentrations of larval hemolymph (10–1280 μg/μL) against the percent inhibition. Data were represented as mean ± SD (*n* = 3).

The total hemolymph antioxidant activities of control and injected *M. domestica* larvae were investigated using the DPPH assay (Figure 4). Results displayed significant radical-scavenging among all LPS-treated samples with respect to the control larval hemolymph. The maximal activity was detected at the 6 h interval, however, the effect decreased time-wise from 6 to 24 h. The inhibitory concentrations (IC_50_) were 611.7 ± 10.41, 63.3 ± 3.51 and 85.7 ± 4.93, 214.3 ± 8.14 µg/mL for control, 6, 12 and 24 h respectively. 

### 3.6. Total Antioxidant Capacity

**Figure 5:**
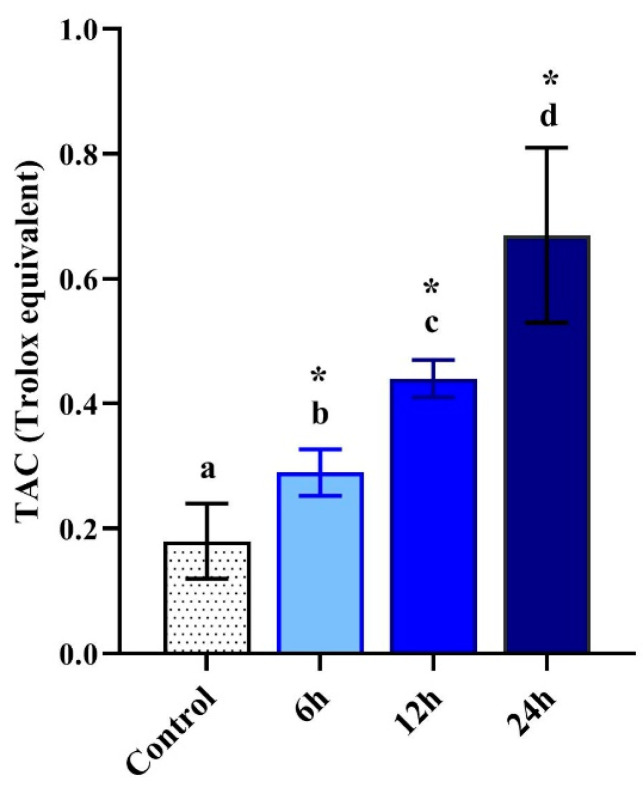
The total antioxidant capacity (TAC) in control and injected *M. domestica* larval hemolymph at 6, 12 and 24 h post-injection with LPS. Different letters indicate significant difference (*p* < 0.05). * indicate high significance with respect to control (*p* < 0.01). Data were expressed as mean ± SD. The experiments were done in triplicate (*n* = 3).

The total antioxidant capacity (TAC) of control and LPS-treated *M. domestica* larval hemolymph displayed a significant (*p* < 0.05) time-dependent elevation in antioxidant activities with 0.18 ± 0.06, 0.29 ± 0.04, 0.44 ± 0.03 and 0.67 ± 0.14 mg TE/g (trolox equivalent) for control, 6, 12 and 24 h respectively (Figure 5).

## 4. Discussion

The antioxidant defense is primarily dependent on the activation of antioxidant enzymes such as superoxide dismutase (SOD), glutathione peroxidase (GPX), catalase (CAT), glutathione S transferase (GSH) and glutathione reductase (GR) [32,72]. The efficiency of the antioxidant system is also linked to the stage of insect ontogenesis meaning that the antioxidant levels are crucial for subsequent developmental stages in larvae [73]. SODs are the major component of insects’ antioxidant enzyme system functioning via the reduction of the concentration of highly reactive superoxide radicals [74,75,76]. The generated free radicals in the body can induce DNA damage [77] and negatively affect other biomolecules in the cell [78].

The hemolymph of insects is a clear fluid, with or without yellow or greenish pigmentation, which makes up 16–40 percent of the insect body weight. The volume and constitutes of the hemolymph differ amongst insect types and their developmental stages. Generally, it contains low molecular weight compounds, such as amino acids, lipids, sugars, inorganic salts and organic acids [79]. Many proteins of the hemolymph help in the protection of the insect from invading microorganisms [80].

The present study demonstrated a significant decrease in lipid peroxidation (MDA), while the antioxidant enzymes, namely SOD and GSH, were increased in the treated larval hemolymph. The injection of LPS induced oxidative stress and subsequent antioxidant responses. The elevated activities of SOD and GSH can be attributed to the insects’ antioxidant defense mechanism [81], which probably validates the early findings showing gradual enzymatic alterations secondary to the infection [82]. Under oxidative stress, the mobilization of the endogenous SOD was increased [83]. Additionally, the enhancement of SOD gene expression during oxidative stress was previously reported [84,85,86]. In this study, the expression of the *M. domestica* superoxide dismutase (*Md*SOD1) gene post-injection with LPS was upregulated relative to the control group of *M. domestica* larvae at 6 and 12 h. The significant upregulation of SOD after LPS injection suggests that the gene transcription was inducible due to the increased level of ROS and subsequent enhancement of SODs expression to balance the effect of ROS [43].

In agreement with Tang et al. [43], the results showed decreased *Md*SOD1-mRNA expression at a 24 h interval in comparison to its upregulated protein level, which might be a consequence of activation of a particular enzymatic pathway at the post-translational level rather than a direct effect on the regulation of antioxidant genes at the transcriptional stage [87]. The turnover of proteins is crucial, whereas, the degradation of mRNA molecules may occur faster than the protein molecules [88], which have a long half-life. The abundance of translated proteins depends on various biological and technical factors [89]. Post-transcriptional alterations, protein stability under certain conditions and mRNA degradation can explain the observed differences in the expression.

The cytotoxic effect of the injected *M. domestica* larval hemolymph was investigated and the results suggested higher cytotoxicity against cancerous MCF-7 cells, while no cytotoxicity was detected on Vero normal cells. The enhanced cytotoxicity may be explained by the activation of internal mechanisms in insects such as proteolytic cascades [90] and the activation of cellular and humoral defense mechanisms that led to the increase in secreted proteins, i.e., defense peptides and stress proteins, into the hemolymph to overcome the induced stress [91]. These peptides displayed many biological activities such as strong antiviral activity against influenza virus [92], antitumor and immunomodulatory properties [27]. Cytotoxicity against cancer cells was also demonstrated [93,94,95,96]. In this work, the enhanced anticancer activity is linked to the elevated antioxidant status, after challenging, which may be attributed to the presence of the defense peptides and stress proteins. Likely, antioxidants can inhibit the tumor formation, its initiation and progression [63], in addition to the induction of apoptosis [97]. The results are consistent with previous studies on insects and their extracts’ anticancer effect, i.e., *M. domestica*, *Lucilia sericata* and *Chrysomya albiceps* extracts on Caco-2 cells [98], *M. domestica* larvae extract on CT26 cells [29] and *L. sericata* and *C. albiceps* larval fluids on HepG-2, MCF-7, HCT-116, A-549 and Hela cell lines [99].

The antioxidant activity of insects has been reported previously in larvae of the blowfly, *Chrysomya megacephala* [100], the aqueous extract of *Vespa affinis* [41]; *Allomyrina dichotoma* rhinoceros beetle larval extracts [81], lucanid beetle, *Serrognathus platymelus* [101] and at various developmental stages of *Protaetia brevitarsis* [102]. Similarly, the present study revealed a significant antioxidant capacity and DPPH radical scavenging of the injected *M. domestica* larval hemolymph. These may be associated with the increased antioxidant capacity of the hemolymph over time similarly to previous reported increase or induction of new proteins to overcome the stress of hydrogen peroxide in *Sarcophaga argyrostoma* larvae [103], and antibacterial proteins in larval hemolymph of *Spodoptera eridania* after LPS injection [104]. Collectively, based on current results, the further purification of hemolymph fractions and the separation of certain compounds and proteins should be performed to investigate the mechanisms of their antioxidant and cytotoxic capacities in vitro and in vivo. Further, the method of LPS-delivery to the larvae will be improved to enable the large scale production of the hemolymph for industrial applications.

## 5. Conclusions

Our study suggests that larval hemolymph of *M. domestica* possesses antioxidant and cytotoxic properties, which were enhanced following the LPS injection. These properties could serve as a promising therapeutic strategy in the management of chronic and degenerative oxidative stress-mediated diseases including cancers.

## Figures and Tables

**Table 1:** Sequences of the primers used in the experiment.

Primer	Accession No.	Reverse	Forward
*Md*SOD1	JF919738	5′CCTCGCCCAAAATCATCTGG′3	5′GCCCAATGATGATGCCTCTC′3
β- actin	JN969088	5′CGGTGGTGGTGAACGAGTAA′3	5′ACACACCAAAATGTGCGACG′3

## References

[1] El-Garawani I., Hassab S., Nabi E., El-Ghandour E. (2017). The Protective Effect of (Foeniculum Vulgare) Oil on Etoposide-Induced Genotoxicity on Male Albino Rats. Eur. J. Pharm. Med. Res..

[2] Saeed M., Amen A., Fahmi A., Garawani I.E., Sayed S. (2014). The Possible Protective Effect of Coriandrum Sativum Seeds Methanolic Extract on Hepato-Renal Toxicity Induced by Sodium Arsenite in Albino Rats. J. Appl. Pharm. Sci..

[3] El-Garawani I., Emam M., Elkhateeb W., El-Seedi H., Khalifa S., Oshiba S., Abou-Ghanima S., Daba G. (2020). In Vitro Antigenotoxic, Antihelminthic and Antioxidant Potentials Based on the Extracted Metabolites from Lichen, Candelariella Vitellina. Pharmaceutics.

[4] El-Garawani I.M., El-Nabi S.E.H., Mohamed A.H., El-Esawy H.M. (2016). Molecular Amelioration of Acacia Arabica Gum on Some Male Reproductive Aspects in Schistosoma Mansoni Infected Mice. Res. J. Pharm. Biol. Chem. Sci..

[5] Ahmed A., Ali M., El-Kholie E., El-Garawani I., Sherif N. (2016). Anticancer Activity of Morus Nigra on Human Breast Cancer Cell Line (MCF-7): The Role of Fresh and Dry Fruit Extracts. J. Biosci. Appl. Res..

[6] Tohamy A.A., El-Garawani I.M., Ibrahim S.R., Abdel Moneim A.E. (2016). The Apoptotic Properties of Salvia Aegyptiaca and Trigonella Foenum-Graecum Extracts on Ehrlich Ascites Carcinoma Cells: The Effectiveness of Combined Treatment. Res. J. Pharm. Biol. Chem. Sci..

[7] El-Garawani I., El Nabi S.H., Nafie E., Almeldin S. (2019). Foeniculum Vulgare and Pelargonium Graveolens Essential Oil Mixture Triggers the Cell Cycle Arrest and Apoptosis in MCF-7 Cells. Anticancer Agents Med. Chem..

[8] El-Garawani I.M., El-Nabi S.H., El-Shafey S., Elfiky M., Nafie E. (2020). Coffea Arabica Bean Extracts and Vitamin C: A Novel Combination Unleashes MCF-7 Cell Death. Curr. Pharm. Biotechnol..

[9] El-Garawani I.M., El-Nabi S.H., Dawoud G.T., Esmail S.M., Abdel Moneim A.E. (2019). Triggering of Apoptosis and Cell Cycle Arrest by Fennel and Clove Oils in Caco-2 Cells: The Role of Combination. Toxicol. Mech. Methods.

[10] Pettit G.R., Meng Y., Herald D.L., Knight J.C., Day J.F. (2005). Antineoplastic Agents. 553. The Texas Grasshopper Brachystola Magna. J. Nat. Prod..

[11] Lee J.-E., Jo D.-E., Lee A.-J., Park H.-K., Youn K., Yun E.-Y., Hwang J.-S., Jun M., Kang B.H. (2015). Hepatoprotective and Anticancer Activities of Allomyrina Dichotoma Larvae. J. Life Sci..

[12] Kim Y.M., Ku M.J., Son Y.-J., Yun J.-M., Kim S.H., Lee S.Y. (2013). Anti-Metastatic Effect of Cantharidin in A549 Human Lung Cancer Cells. Arch. Pharm. Res..

[13] Ali M.M., Arumugam Sarasa B.A. (2011). Effect of Crude Extract of Bombyx Mori Coccoons in Hyperlipidemia and Atherosclerosis. J. Ayurveda Integr. Med..

[14] Zhou Y.L., Wang R., Feng X., Zhao X. (2014). Preventive Effect of Insect Tea against Reserpine-Induced Gastric Ulcers in Mice. Exp. Ther. Med..

[15] Khan M.S., Singh M., Khan M.A., Arya D.S., Ahmad S. (2014). Scientific Validation of Cardioprotective Attribute by Standardized Extract of Bombyx Mori against Doxorubicin-Induced Cardiotoxicity in Murine Model. Excli J..

[16] Prakash S., Bhargava H.R. (2014). Apis Cerana Bee Venom: It’s Anti-Diabetic and Anti-Dandruff Activity against Malassezia Furfur. World Appl. Sci. J..

[17] Józefiak A., Engberg R.M. (2017). Insect Proteins as a Potential Source of Antimicrobial Peptides in Livestock Production. A Review. J. Anim. Feed Sci..

[18] Wu Q., Patočka J., Kuča K. (2018). Insect Antimicrobial Peptides, a Mini Review. Toxins.

[19] Tang J.J., Fang P., Xia H.L., Tu Z.C., Hou B.Y., Yan Y.M., Di L., Zhang L., Cheng Y.X. (2015). Constituents from the Edible Chinese Black Ants (Polyrhachis Dives) Showing Protective Effect on Rat Mesangial Cells and Anti-Inflammatory Activity. Food Res. Int..

[20] Yan Y.M., Li L.J., Qin X.C., Lu Q., Tu Z.C., Cheng Y.X. (2015). Compounds from the Insect Blaps Japanensis with COX-1 and COX-2 Inhibitory Activities. Bioorganic Med. Chem. Lett..

[21] Tiveron A.P., Rosalen P.L., Franchin M., Lacerda R.C.C., Bueno-Silva B., Benso B., Denny C., Ikegaki M., De Alencar S.M. (2016). Chemical Characterization and Antioxidant, Antimicrobial, and Anti-Inflammatory Activities of South Brazilian Organic Propolis. PLoS ONE.

[22] Shittu O.K., Bashir L., Isaac O.O. (2014). Effects of Methanol Extract of Musca Domestica Larvae on Antioxidants Enzymes in T. Brucei Infected Rats. Niger. J. Biochem. Mol. Biol..

[23] Nmorsi O.P.G., Ukwandu N.C.D., Agbozele G.E. (2006). Detection of Some Gastrointestinal Parasites from Four Synanthropic Flies in Ekpoma, Nigeria. J. Vector Borne Dis..

[24] An C., Li D., Du R. (2004). Analysis of Antibacterial-Relative Proteins and Peptides in Housefly Larvae. Wei Sheng Yan Jiu.

[25] Guo G., Tao R., Li Y., Ma H., Xiu J., Fu P., Wu J. (2017). Identification and Characterization of a Novel Antimicrobial Protein from the Housefly Musca Domestica. Biochem. Biophys. Res. Commun..

[26] Ok S., Olayemi I.K., Omalu I.C., Adeniyi A.K. (2013). Anti-Plasmodial properties of methanolic extract of musca domestica maggot on P. berghei—Infected mice. Int. J. Biol. Pharm. Allied Sci..

[27] Sun H.X., Chen L.Q., Zhang J., Chen F.Y. (2014). Anti-Tumor and Immunomodulatory Activity of Peptide Fraction from the Larvae of Musca Domestica. J. Ethnopharmacol..

[28] Zhao R.J., Zhang Q.H., Li F.D. (2007). The Effection of Antimicrobial Peptides Extracted from Adult Housefly on Tumour C. Chin. J. Vector Biol. Control.

[29] Hou L., Shi Y., Zhai P., Le G. (2007). Antibacterial Activity and in Vitro Anti-Tumor Activity of the Extract of the Larvae of the Housefly (Musca Domestica). J. Ethnopharmacol..

[30] Boman H.G. (1995). Peptide Antibiotics and Their Role in Innate Immunity. Annu. Rev. Immunol..

[31] Bulet P., Hetru C., Dimarcq J.L., Hoffmann D. (1999). Antimicrobial Peptides in Insects; Structure and Function. Dev. Comp. Immunol..

[32] Barbehenn R.V. (2002). Gut-Based Antioxidant Enzymes in a Polyphagous and a Graminivorous Grasshopper. J. Chem. Ecol..

[33] Felton G.W., Summers C.B. (1995). Antioxidant Systems in Insects. Arch. Insect Biochem. Physiol..

[34] Zielińska E., Baraniak B., Karaś M., Rybczyńska K., Jakubczyk A. (2015). Selected Species of Edible Insects as a Source of Nutrient Composition. Food Res. Int..

[35] Zielińska E., Karaś M., Jakubczyk A. (2017). Antioxidant Activity of Predigested Protein Obtained from a Range of Farmed Edible Insects. Int. J. Food Sci. Technol..

[36] Gobbetti M., Stepaniak L., De Angelis M., Corsetti A., Di Cagno R. (2002). Latent Bioactive Peptides in Milk Proteins: Proteolytic Activation and Significance in Dairy Processing. Crit. Rev. Food Sci. Nutr..

[37] Saiga A., Tanabe S., Nishimura T. (2003). Antioxidant Activity of Peptides Obtained from Porcine Myofibrillar Proteins by Protease Treatment. J. Agric. Food Chem..

[38] Zhang M., Mu T.H., Wang Y.B., Sun M.J. (2012). Evaluation of Free Radical-Scavenging Activities of Sweet Potato Protein and Its Hydrolysates as Affected by Single and Combination of Enzyme Systems. Int. J. Food Sci. Technol..

[39] Karaś M., Jakubczyk A., Szymanowska U., Złotek U., Zielińska E. (2017). Digestion and Bioavailability of Bioactive Phytochemicals. Int. J. Food Sci. Technol..

[40] Di Mattia C., Battista N., Sacchetti G., Serafini M. (2019). Antioxidant Activities in Vitro of Water and Liposoluble Extracts Obtained by Different Species of Edible Insects and Invertebrates. Front. Nutr..

[41] Dutta P., Dey T., Manna P., Kalita J. (2016). Antioxidant Potential of *Vespa affinis* L., a Traditional Edible Insect Species of North East India. PLoS ONE.

[42] Mahmoud S., Moselhy W., El-Khashab L.A., Seufi A.M. (2018). Identification and Molecular Characterisation of a Novel Manganese Superoxide Dismutase Gene from Flesh Fly Larvae, Sarcophaga Argyrostoma (Diptera: Sarcophagidae). Afr. Entomol..

[43] Tang T., Huang D.-W., Zhou C.-Q., Li X., Xie Q.-J., Liu F.-S. (2012). Molecular Cloning and Expression Patterns of Copper/Zinc Superoxide Dismutase and Manganese Superoxide Dismutase in Musca Domestica. Gene.

[44] Večeřa J., Krishnan N., Alquicer G., Kodrík D., Socha R. (2007). Adipokinetic Hormone-Induced Enhancement of Antioxidant Capacity of Pyrrhocoris Apterus Hemolymph in Response to Oxidative Stress. Comp. Biochem. Physiol. C Toxicol. Pharmacol..

[45] Sun Y., Wu Y., Wang Z., Chen J., Yang Y., Dong G. (2020). Dandelion Extract Alleviated Lipopolysaccharide-Induced Oxidative Stress through the Nrf2 Pathway in Bovine Mammary Epithelial Cells. Toxins (Basel).

[46] Li L., Wang H.H., Nie X.T., Jiang W.R., Zhang Y.S. (2019). Sodium Butyrate Ameliorates Lipopolysaccharide-Induced Cow Mammary Epithelial Cells from Oxidative Stress Damage and Apoptosis. J. Cell. Biochem..

[47] Magdaleno F., Blajszczak C.C., Nieto N. (2017). Key Events Participating in the Pathogenesis of Alcoholic Liver Disease. Biomolecules.

[48] Gomes J.M.G., de Assis Costa J., de Cássia Gonçalves Alfenas R. (2017). Metabolic Endotoxemia and Diabetes Mellitus: A Systematic Review. Metab. Clin. Exp..

[49] Choi Y.S., Lee K.S., Yoon H.J., Kim I., Sohn H.D., Jin B.R. (2006). Bombus Ignitus Cu,Zn Superoxide Dismutase (SOD1): CDNA Cloning, Gene Structure, and up-Regulation in Response to Paraquat, Temperature Stress, or Lipopolysaccharide Stimulation. Comp. Biochem. Physiol. B Biochem. Mol. Biol..

[50] Parusel R., Steimle A., Lange A., Schäfer A., Maerz J.K., Bender A., Frick J.S. (2017). An Important Question: Which LPS Do You Use?. Virulence.

[51] Homa J., Stalmach M., Wilczek G., Kolaczkowska E. (2016). Effective Activation of Antioxidant System by Immune-Relevant Factors Reversely Correlates with Apoptosis of Eisenia Andrei Coelomocytes. J. Comp. Physiol. B Biochem. Syst. Environ. Physiol..

[52] Chu F.J., Jin X.B., Xu Y.Y., Ma Y., Li X.B., Lu X.M., Liu W.B., Zhu J.Y. (2013). Inflammatory Regulation Effect and Action Mechanism of Anti-Inflammatory Effective Parts of Housefly (Musca Domestica) Larvae on Atherosclerosis. Evid. Based Complement. Altern. Med..

[53] Wu G., Yi Y., Lv Y., Li M., Wang J., Qiu L. (2015). The Lipopolysaccharide (LPS) of Photorhabdus Luminescens TT01 Can Elicit Dose- and Time-Dependent Immune Priming in Galleria Mellonella Larvae. J. Invertebr. Pathol..

[54] Stadtman E.R. (2006). Protein Oxidation and Aging. Free Radic. Res..

[55] Ali S.S., Kasoju N., Luthra A., Singh A., Sharanabasava H., Sahu A., Bora U. (2008). Indian Medicinal Herbs as Sources of Antioxidants. Food Res. Int..

[56] Sosa V., Moliné T., Somoza R., Paciucci R., Kondoh H., LLeonart M.E. (2013). Oxidative Stress and Cancer: An Overview. Ageing Res. Rev..

[57] Baynes J.W. (1991). Role of Oxidative Stress in Development of Complications in Diabetes. Diabetes.

[58] Torres-Fuentes C., Alaiz M., Vioque J. (2011). Affinity Purification and Characterisation of Chelating Peptides from Chickpea Protein Hydrolysates. Food Chem..

[59] Karaś M., Baraniak B., Rybczyńska K., Gmiński J., Gaweł-Bęben K., Jakubczyk A. (2015). The Influence of Heat Treatment of Chickpea Seeds on Antioxidant and Fibroblast Growth-Stimulating Activity of Peptide Fractions Obtained from Proteins Digested under Simulated Gastrointestinal Conditions. Int. J. Food Sci. Technol..

[60] Carrasco-Castilla J., Hernández-Álvarez A.J., Jiménez-Martínez C., Jacinto-Hernández C., Alaiz M., Girón-Calle J., Vioque J., Dávila-Ortiz G. (2012). Antioxidant and Metal Chelating Activities of Phaseolus Vulgaris L. Var. Jamapa Protein Isolates, Phaseolin and Lectin Hydrolysates. Food Chem..

[61] Tak P.P., Zvaifler N.J., Firestein G.S., Greene D.R. (2000). Rheumatoid Arthritis and P53: How Oxidative Stress, Might Alter the Course of Inflammatory Diseases. Immunol. Today.

[62] Manna P., Bhattacharyya S., Das J., Ghosh J., Sil P.C. (2011). Phytomedicinal Role of Pithecellobium Dulce against Ccl4 -Mediated Hepatic Oxidative Impairments and Necrotic Cell Death. Evid. Based Complement. Altern. Med..

[63] O‘Brien’ P.J. (1994). Antioxidants and Cancer: Molecular Mechanism. Free Radicals in Diagnostic Medicine.

[64] Hashem H.O., Youssef N.S. (1991). Developmental Changes Induced by Methanolic Extracts of Leaves and Fruits of Melia Azedarach L. on the House Fly Musca Domestica Vicina MACQ. J. Egypt. Ger. Soc. Zool..

[65] Marklund S., Marklund G. (1974). Involvement of the Superoxide Anion Radical in the Autoxidation of Pyrogallol and a Convenient Assay for Superoxide Dismutase. Eur. J. Biochem..

[66] Ellman G.L. (1959). Tissue Sulfhydryl Groups. Arch. Biochem. Biophys..

[67] Mihara M., Uchiyama M. (1978). Determination of Malonaldehyde Precursor in Tissues by Thiobarbituric Acid Test. Anal. Biochem..

[68] Laemmli U.K. (1970). Cleavage of Structural Proteins during the Assembly of the Head of Bacteriophage T4. Nature.

[69] Burnette W.N. (1981). “Western Blotting”: Electrophoretic Transfer of Proteins from Sodium Dodecyl Sulfate-Polyacrylamide Gels to Unmodified Nitrocellulose and Radiographic Detection with Antibody and Radioiodinated Protein A. Anal. Biochem..

[70] Wang Y., Wang L., Zhu Z., Ma W., Lei C. (2012). The Molecular Characterization of Antioxidant Enzyme Genes in Helicoverpa Armigera Adults and Their Involvement in Response to Ultraviolet-A Stress. J. Insect Physiol..

[71] Shimada K., Fujikawa K., Yahara K., Nakamura T. (1992). Antioxidative Properties of Xanthone on the Auto Oxidation of Soybean in Cylcodextrin Emulsion. J. Agric. Food Chem..

[72] Meng J.Y., Zhang C.Y., Zhu F., Wang X.P., Lei C.L. (2009). Ultraviolet Light-Induced Oxidative Stress: Effects on Antioxidant Response of Helicoverpa Armigera Adults. J. Insect Physiol..

[73] Jovanović-Galović A., Blagojević D.P., Grubor-Lajšić G., Worland R., Spasić M.B. (2004). Role of Antioxidant Defense during Different Stages of Preadult Life Cycle in European Corn Borer (Ostrinia Nubilalis, Hubn.): Diapause and Metamorphosis. Arch. Insect Biochem. Physiol..

[74] Pritsos C.A., Ahmad S., Elliott A.J., Pardini R.S. (1990). Antioxidant Enzyme Level Response to Prooxidant Allelochemicals in Larvae of the Southern Armyworm Moth, Spodoptera Eridania. Free Radic. Res..

[75] Wang Y., Oberley L.W., Murhammer D.W. (2001). Antioxidant Defense Systems of Two Lipidopteran Insect Cell Lines. Free Radic. Biol. Med..

[76] Weirich G.F., Collins A.M., Williams V.P. (2002). Antioxidant Enzymes in the Honey Bee, Apis Mellifera. Apidologie.

[77] Lee K.G., Mitchell A.E., Shibamoto T. (2000). Determination of Antioxidant Properties of Aroma Extracts from Various Beans. J. Agric. Food Chem..

[78] Dreher D., Junod A.F. (1996). Role of Oxygen Free Radicals in Cancer Development. Eur. J. Cancer.

[79] Wyatt G.R. (1961). The Biochemistry of Insect Hemolymph. Annu. Rev. Entomol..

[80] Lemaitre B., Hoffmann J. (2007). The Host Defense of Drosophila Melanogaster. Annu. Rev. Immunol..

[81] Suh H.J., Kim S.R., Lee K.S., Park S., Kang S.C. (2010). Antioxidant Activity of Various Solvent Extracts from Allomyrina Dichotoma (Arthropoda: Insecta) Larvae. J. Photochem. Photobiol. B Biol..

[82] Pentreath V.W., Kennedy P.G.E. (2009). Pathogenesis of Human African Trypanosomiasis. The trypanosomiases.

[83] Atalay M., Laaksonen D.E., Khanna S., Kaliste-Korhonen E., Hänninen O., Sen C.K. (2000). Vitamin E Regulates Changes in Tissue Antioxidants Induced by Fish Oil and Acute Exercise. Med. Sci. Sports Exerc..

[84] Whiteside C., Hassan H.M. (1987). Induction and Inactivation of Catalase and Superoxide Dismutase of Escherichia Coli by Ozone. Arch. Biochem. Biophys..

[85] Farr S.B., Kogoma T. (1991). Oxidative Stress Responses in Escherichia Coli and Salmonella Typhimurium. Microbiol. Rev..

[86] Wang J.L., Liu X.S., Zhang Q., Zhao H.B., Wang Y.F. (2012). Expression Profiles of Six Novel C-Type Lectins in Response to Bacterial and 20E Injection in the Cotton Bollworm (Helicoverpa Armigera). Dev. Comp. Immunol..

[87] Večeřa J., Krishnan N., Mithöfer A., Vogel H., Kodrík D. (2012). Adipokinetic Hormone-Induced Antioxidant Response in Spodoptera Littoralis. Comp. Biochem. Physiol. C Toxicol. Pharmacol..

[88] Wu G., Nie L., Zhang W. (2008). Integrative Analyses of Posttranscriptional Regulation in the Yeast Saccharomyces Cerevisiae Using Transcriptomic and Proteomic Data. Curr. Microbiol..

[89] Maier T., Güell M., Serrano L. (2009). Correlation of MRNA and Protein in Complex Biological Samples. Febs Lett..

[90] Hoffmann J.A., Reichhart J.M., Hetru C. (1996). Innate Immunity in Higher Insects. Curr. Opin. Immunol..

[91] Grizanova E.V., Dubovskiy I.M., Whitten M.M.A., Glupov V.V. (2014). Contributions of Cellular and Humoral Immunity of Galleria Mellonella Larvae in Defence against Oral Infection by Bacillus Thuringiensis. J. Invertebr. Pathol..

[92] Wang F., Ai H., Lei C. (2013). In Vitro Anti-Influenza Activity of a Protein-Enriched Fraction from Larvae of the Housefly (Musca Domestica). Pharm. Biol..

[93] Moore A.J., Devine D.A., Bibby M.C. (1994). Preliminary Experimental Anticancer Activity of Cecropins. Pept. Res..

[94] Mader J.S., Hoskin D.W. (2006). Cationic Antimicrobial Peptides as Novel Cytotoxic Agents for Cancer Treatment. Expert Opin. Investig. Drugs.

[95] Hoskin D.W., Ramamoorthy A. (2008). Studies on Anticancer Activities of Antimicrobial Peptides. Biochim. Biophys. Acta Biomemb..

[96] Berge G., Eliassen L.T., Camilio K.A., Bartnes K., Sveinbjørnsson B., Rekdal Ø. (2010). Therapeutic Vaccination against a Murine Lymphoma by Intratumoral Injection of a Cationic Anticancer Peptide. Cancer Immunol. Immunother..

[97] Tsai J.C., Jain M., Hsieh C.M., Lee W.S., Yoshizumi M., Patterson C., Perrella M.A., Cooke C., Wang H., Haber E. (1996). Induction of Apoptosis by Pyrrolidinedithiocarbamate and N-Acetylcysteine in Vascular Smooth Muscle Cells. J. Biol. Chem..

[98] Hasaballah A., Shehata A., Shehab A. (2019). Antioxidant and Anticancer Activities of Some Maggots Methanol Extracts. Egypt. Acad. J. Biol. Sci. Aentomol..

[99] Shehata A., Mehany A., El-Sheikh T. (2016). Excretion/Secretion of Lucilia Sericata and Chrysomya Albiceps (Diptera: Calliphoridae) Maggots as Potential Anticancer Agent and Kinases Inhibitor. N. Y. Sci. J..

[100] Song C., Yu H., Zhang M., Yang Y., Zhang G. (2013). Physicochemical Properties and Antioxidant Activity of Chitosan from the Blowfly Chrysomya Megacephala Larvae. Int. J. Biol. Macromol..

[101] Suh H.J., Kim S.R., Hwang J.S., Kim M.J., Kim I. (2011). Antioxidant Activity of Aqueous Methanol Extracts from the Lucanid Beetle, Serrognathus Platymelus Castanicolor Motschulsky (Coleoptera: Lucanidae). J. Asia Pac. Entomol..

[102] Suh H.-J., Kang S.C. (2012). Antioxidant Activity of Aqueous Methanol Extracts of Protaetia Brevitarsis Lewis (Coleoptera: Scarabaedia) at Different Growth Stages. Nat. Prod. Res..

[103] Mahmoud S.H., Moselhy W.A., El-Khashab L.A.A., Abdelbaset B.Z., Seufi A.M. (2016). Variations induced in electrophoretic pattern of haemolymph proteins of flesh fly, sarcophaga argyrostoma (diptera: Sarcophagidae) larvae challenged with hydrogen peroxide. Int. J. Adv. Res..

[104] Anderson R.S., Cook M.L. (1979). Induction of Lysozymelike Activity in the Hemolymph and Hemocytes of an Insect, Spodoptera Eridania. J. Invertebr. Pathol..

